# Clinical and Analytical Performance of an Automated Serological Test That Identifies S1/S2-Neutralizing IgG in COVID-19 Patients Semiquantitatively

**DOI:** 10.1128/JCM.01224-20

**Published:** 2020-08-24

**Authors:** Fabrizio Bonelli, Antonella Sarasini, Claudia Zierold, Mariella Calleri, Alice Bonetti, Chiara Vismara, Frank A. Blocki, Luca Pallavicini, Alberto Chinali, Daniela Campisi, Elena Percivalle, Anna Pia DiNapoli, Carlo Federico Perno, Fausto Baldanti

**Affiliations:** a DiaSorin, S.p.A., Saluggia, Italy; b Molecular Virology Unit, Fondazione Istituto di Ricovero e Cura a Carattere Scientifico (IRCCS) Policlinico San Matteo, Pavia, Italy; c Department of Clinical, Surgical Diagnostic and Pediatric Sciences, University of Pavia, Pavia, Italy; d Department of Laboratory Medicine, ASST Niguarda Hospital, University of Milan, Milan, Italy; UNC School of Medicine

**Keywords:** CLIA, COVID-19, SARS-CoV-2, diagnostics, immunoassays, immunoserology, neutralization assay, neutralizing antibodies, spike

## Abstract

In the coronavirus (CoV) disease 2019 (COVID-19) pandemic, highly selective serological testing is essential to define exposure to severe acute respiratory syndrome CoV 2 (SARS-CoV-2). Many tests have been developed, yet with variable speeds to first results, and are of unknown quality, particularly when considering the prediction of neutralizing capacity. The LIAISON SARS-CoV-2 S1/S2 IgG assay was designed to measure antibodies against the SARS-CoV-2 native S1/S2 proteins in a standardized automated chemiluminescence assay. The clinical and analytical performances of the test were validated in an observational study using residual samples (>1,500) with a positive or negative COVID-19 diagnosis. The LIAISON SARS-CoV-2 S1/S2 IgG assay proved to be highly selective and specific and offered semiquantitative measures of serum or plasma levels of anti-S1/S2 IgG with neutralizing activity. The assay’s diagnostic sensitivities were 91.3% and 95.7% at >5 or ≥15 days from diagnosis, respectively, and 100% when assessed against a neutralizing assay. The assay’s specificity ranged between 97% and 98.5%. The average imprecision of the assay was a <5% coefficient of variation. Assay performance at 2 different cutoffs was evaluated to optimize predictive values. The automated LIAISON SARS-CoV-2 S1/S2 IgG assay brings efficient, sensitive, specific, and precise serological testing to the laboratory, with the capacity to test large amounts of samples per day; first results are available within 35 min, with a throughput of 170 tests/hour. The semiquantitative results provided by the test also associate with the presence of neutralizing antibodies and may provide a useful tool for the large-scale screening of convalescent-phase plasma for safe therapeutic use.

## Abstract (precis)

In the coronavirus (CoV) disease 2019 (COVID-19) pandemic, highly selective serological testing is essential to define exposure to severe acute respiratory syndrome CoV 2 (SARS-CoV-2). Many tests have been developed, yet with variable speeds to first results, and are of unknown quality, particularly when considering the prediction of neutralizing capacity. The LIAISON SARS-CoV-2 S1/S2 IgG assay was designed to measure antibodies against the SARS-CoV-2 native S1/S2 proteins in a standardized automated chemiluminescence assay.

## INTRODUCTION

Severe acute respiratory syndrome coronavirus 2 (SARS-CoV-2), the virus responsible for the CoV disease 2019 (COVID-19) pandemic, has spread at an alarming rate since the first case, which tracked back to mid-November of 2019 in Wuhan, China (1). Contraction and subsequent transmission accrue most prevalently from community exposure, from nonhuman exposure, or among relatives living in proximity to symptomatic or asymptomatic infected individuals. Due to the lack of readily available diagnostics, inferior means of infection control, or the inability to triage and isolate both acute and suspected cases as a consequence of space limitations, the COVID-19 pandemic, in placing increasingly excessive demands on the global health care network, has unveiled a number of critical limitations (2).

No safe vaccines have been developed for SARS-CoV-2 infections to date, and the lack of currently available effective antiviral therapies, in spite of years of ongoing research, are certainly hampering efforts to combat this pandemic. In addition, the current molecularly based diagnostic tools utilized to diagnose infection, though serving adequately as the only means available, are not suitable for mass screening, and though many serological assays have been developed, no scientific data are available to authenticate their effectiveness. Finally, once the World Health Organization (WHO) recognized the SARS-CoV-2 outbreak as a public health emergency of international concern on 30 January 2020, efforts have been hampered internationally, nationally, and locally by a lack of coordinated guidance to properly inform public policymakers and a lack of ready access to accurate and rapid testing.

There is a knowledge gap in understanding the efficiency of community transmission of SARS-CoV-2, including the contribution of mild or asymptomatic cases (1). As of 5 May 2020, global cases have surpassed 3 million, with 254,592 registered deaths (3). Global mortality consequent to SARS-CoV-2 infection is running at 7.0%, with national rates for France, Italy, Spain, the United Kingdom, Germany, and the United States running at 14.9%, 13.7%, 11.7%, 15.0%, 4.2%, and 5.8%, respectively (4).

In view of these daunting numbers, effective, sensitive, and specific means for the identification and laboratory confirmation of SARS-CoV-2 infection are urgently needed. In response to these needs, DiaSorin has developed a highly sensitive, specific, automated, and contained chemiluminescence serological assay for the detection of SARS-CoV-2 spike protein-specific antibodies with neutralizing potential from serum or plasma to be used in diagnostic, epidemiological, and vaccine evaluation studies. Specifically, it is envisioned that the test may be used (i) to screen infected health care workers and the general population for recovery and/or past exposure; (ii) in epidemiological studies characterizing the demographics of viral spread and the efficacy of containment measures directed toward SARS-CoV-2 at the local, national, and international levels; (iii) to screen convalescent-phase sera for both therapeutic and prophylactic use, and (iv) to evaluate vaccine effectiveness in clinical studies.

## MATERIALS AND METHODS

### Assay format.

The LIAISON SARS-CoV-2 S1/S2 IgG chemiluminescence assay is a recently developed assay from DiaSorin designed to detect IgG antibodies in the serum or plasma of subjects and patients exposed to SARS-CoV-2. The assay consists of paramagnetic microparticles (PMPs) coated with S1 and S2 fragments of the viral surface spike protein (5). Recombinant fusion antigens were expressed in human cells (HEK-293) to ensure proper folding, oligomer formation, and glycosylation, providing capture moieties more similar to those of the viral spike proteins, as processed by natural cellular cleavage (6–8); this distinguishes the DiaSorin chemiluminescence immunoassay (CLIA) from commonly used enzyme-linked immunoassays (ELISAs) where the antigens are presented on plastic plate surfaces and are susceptible to significant denaturation consequent to passive adsorption to these hydrophobic surfaces (9, 10). Distally biotinylated-S1 and biotinylated-S2 proteins were tethered to the surfaces of paramagnetic particles coated with streptavidin to ensure optimal presentation of both S1 and S2 for access and recognition by specific immunoglobulin within pathologic serum samples.

The automated assay format consists of a first incubation step (10 min) of S1/S2-coated PMPs with a patient sample (20 μl of either plasma or serum) in assay buffer to allow the binding of IgG in the sample specific to the antigens, followed by a wash step to remove unbound materials. Next, ABEI [*N*-(4-aminobutyl)-*N*-ethyl-isoluminol]-labeled polyclonal goat anti-human IgG are added to the PMPs and further incubated for 8 min. After a final wash cycle, starter reagents are added, and emitted relative light units (RLU) proportional to the sample’s anti-S1/S2 IgG levels are converted to arbitrary units (AU)/milliliter based on a standardized master curve. The automated assay is standardized based on a pool of patient samples with high S1/S2 IgG titers. First results are available within 35 min, and the throughput is 170 tests/hour.

### Analytical assay performance.

A 5-day precision study according to CLSI EP5-A3 guidelines was performed using a panel of 6 plasma samples, prepared by either spiking or diluting as necessary to obtain negative, slightly positive, and moderately positive samples. The panel samples were tested with the LIAISON SARS-CoV-2 S1/S2 IgG assay in 6 replicates per run and 3 runs per day for 5 operating days on one LIAISON XL analyzer (*n* = 90).

A cross-reactivity study was performed to evaluate other SARS viruses (human CoV-229E [HCoV-229E], HCoV-HKU1, HCoV-OC43, and untyped HCoV), and samples from patients with conditions caused by other viruses, other organisms, or atypical immune system activity (nuclear autoantibodies, herpesvirus B, herpesvirus C, influenza A virus, influenza B virus, respiratory syncytial virus, Borrelia burgdorferi, Mycoplasma pneumoniae, Epstein-Barr virus, cytomegalovirus, herpes simplex viruses 1 and 2, human anti-mouse antibodies, parvovirus B19, rheumatoid factor, rubella virus, and varicella zoster virus). Samples for the evaluation were collected before October 2019, prior to the COVID-19 pandemic. In addition, samples with potentially interfering factors, such as triglycerides, hemoglobin, bilirubin, cholesterol, acetaminophen, ibuprofen, and biotin, were assessed with the LIAISON SARS-CoV-2 S1/S2 IgG assay according to CLSI guidelines.

### SARS-CoV-2 microneutralization assay (NT assay).

A neutralizing assay described elsewhere was used to determine the neutralization titer against SARS-CoV-2 (26). Briefly, 50 μl of diluted serum (4-fold serial dilutions from 1:10 to 1:640) were added to an equal volume of viral suspension (tissue culture infectious dose of 50 from a SARS-CoV-2 strain isolated from a symptomatic patient), incubated, and then combined with Vero-E6 cells. After incubation, the cells were stained with Gram’s crystal violet solution. Wells were scored to evaluate the degree of cytopathic effect compared to that of viral controls. The neutralizing titer was the maximum dilution evidencing a reduction of 90% of the cytopathic effect. In this study, a titer of ≥1:40 was considered positive. This test was used to confirm positive serological samples used in the clinical studies and to determine the neutralization effectiveness of the samples for the identification of convalescent donors living in the first Italian Red Zone (Percivalle et al., submitted for publication).

### Clinical samples.

This observational study used deidentified fresh or frozen residual samples collected between 2011 and April 2020 at the Policlinico San Matteo in Pavia, Italy, and at the Niguarda Hospital in Milan, Italy. Sample groups included (i) paired samples (admission and discharge) from non-intensive care unit (ICU), hospitalized patients with moderate symptoms affected by COVID-19 (confirmed by real-time PCR [RT-PCR]; *n* = 31); (ii) sets of samples (admission and follow-up samples) from patients affected by COVID-19 and hospitalized in the ICU with severe symptoms (confirmed by RT-PCR; *n* = 16); (iii) samples from patients affected by COVID-19 and hospitalized in the ICU with severe symptoms (confirmed by RT-PCR; *n* = 21); (iv) samples from outpatients affected by COVID-19 testing positive by RT-PCR (*n* = 37); (v) samples from subjects collected before the outbreak of COVID-19 (lab routine, 2011; *n* = 1,140); (vi) samples from subjects not infected by SARS-CoV-2 but affected by other coronaviruses, i.e., HCoV-229E, HCoV-HKU1, HCoV-OC43, or an untyped HCoV strain (*n* = 10); (vii) samples from subjects testing negative by RT-PCR (*n* = 50); (viii) samples negative by the SARS-CoV-2 NT assay, described in Materials and Methods, collected from subjects during the outbreak (*n* = 180); and (ix) NT assay-positive samples collected from subjects during the outbreak (*n* = 124).

A summary of the patient groups is shown in Table 1. Pertinent additional information included sample collection time, days from diagnosis (hospital or ICU admission), and severity of symptoms (mild, moderate, severe). The protocol for this study (deidentified remainders) was determined to be exempt under existing ethics committee regulations.

**TABLE 1:** Summary of clinical sample types assessed in this observational study

Group	No. of clinical samples (*n* = 1,609)	Hospital stay	ICU stay	SARS-CoV-2 RT-PCR positive	NT^*a*^ testing	Serial samples
Paired COVID-19	31	Yes	No	Yes	No	Yes, admission (1st) and discharge (2nd)
ICU serial	16	Yes	Yes	Yes	No	Yes
ICU	21	Yes	Yes	Yes	No	No
Outpatient positive	37	No	No	Yes	No	No
Pre-COVID-19 negative	1,140	No	No	Not tested	No	No
Infected with other CoVs	10	No	No	Not tested	No	No
COVID-19 negative	50	No	No	No	No	No
NT negative	180	No	No	Unknown	Negative	No
NT positive	124	Unknown	Unknown	Unknown	Positive	No

a NT, neutralization.

### Diagnosis.

Diagnosis was based on results from routine RT-PCR used in the clinical evaluation (11), as well as NT assay results. Samples not infected by SARS-CoV-2 but affected by other coronaviruses were classified by sequencing.

### Statistical analyses.

The statistical program R 3.5 and MedCalc 19.2 were utilized for all analyses presented. A preliminary exploration using Box-Cox methodology suggested that statistical analyses be done on the logarithmic scale due to skewed distribution of the measurements.

Data supporting figures and tables in this paper will be made fully available upon request.

## RESULTS

### Sample characteristics.

**FIG 1:**
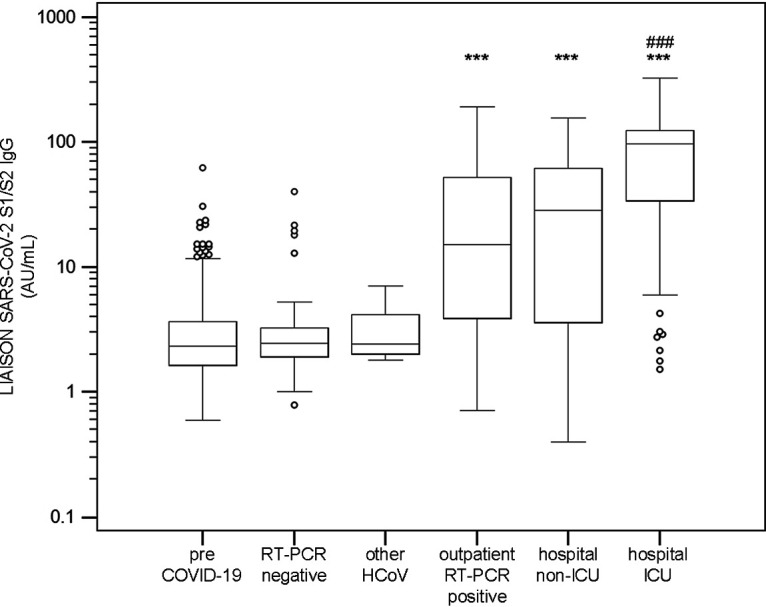
Distribution of SARS-CoV-2 S1/S2 IgG levels in various patient groups. All the patient groups categorized as positive (RT-PCR positive, hospitalized, and in the ICU) are significantly different from the negative groups (RT-PCR negative, pre-COVID-19, and infected with other HCoVs) at a *P* of <0.0001 (***), as determined by a pairwise *t* test comparison with Bonferroni multiplicity adjustment. The ICU patient group is significantly different from the hospitalized patient group (###, *P* < 0.0001).

Clinical assessment of the LIAISON SARS-CoV-2 S1/S2 IgG assay was performed using various sample groups (Fig. 1). All the patient groups categorized as positive for COVID-19 were significantly different from the negative groups, with a *P* of <0.0001 as determined by a pairwise *t* test comparison with Bonferroni multiplicity adjustment. Median S1/S2 IgG levels were 96.3 AU/ml (95% confidence intervals [95% CI], 85.8 to 108.0 AU/ml; *n* = 64), 28.6 AU/ml (95% CI, 10.6 to 45.1 AU/ml; *n* = 67), and 15.5 AU/ml (95% CI, 5.7 to 32.2 AU/ml; *n* = 80) for intensive care unit (ICU) patients, non-ICU hospitalized patients, and RT-PCR-positive outpatients, respectively. Median levels of negative samples were 2.3 AU/ml (95% CI, 2.2 to 2.4 AU/ml; *n* = 1,140), 2.4 AU/ml (95% CI, 2.1 to 2.9 AU/ml; *n* = 50), and 2.2 AU/ml (95% CI, 1.8 to 4.6 AU/ml; *n* = 10) for pre-COVID-19, RT-PCR-negative, and other coronavirus (non-SARS-Cov-2) subjects, respectively. In addition, the ICU patient group had significantly higher levels of S1/S2 IgG than the non-ICU, hospitalized patient group (*P* < 0.0001). Table 2 further dissects the temporal component of this distribution, where early samples with low levels of S1/S2 IgG present a low positive predictive agreement (PPA) with RT-PCR results at a time ≤5 days from diagnosis (33.3%) that increases to 95.7% at ≥15 days from diagnosis.

**TABLE 2:** Longitudinal assessment of positive predictive agreement with an RT-PCR diagnosis for COVID-19 patients^*a*^

No. of days from diagnosis	No. of subjects						Cumulative no. of S1/S2 IgG^+^ subjects/total	PPA (95%CI)
	First serial measurement		Second serial measurement		Third serial measurement			
	Total	S1/S2 IgG^+^	Total	S1/S2 IgG^+^	Total	S1/S2 IgG^+^		
≤5	84	28					28/84	33.3 (23.4–44.5)
6–14	7	7	71	62	1	1	70/79	88.6 (79.5–94.7)
≥15	13	13	12	12	22	20	45/47	95.7 (85.5–99.5)
Total no. of subjects	104		83		23			

a Serial samples from 104 COVID-19 patients positive by RT-PCR admitted to the hospital or ICU were tested with the LIAISON SARS-CoV-2 S1/S2 IgG assay. A value of 9 AU/ml was used as the cutoff for positivity. All samples in the same time brackets across measurements are from different patients, except for 3 samples from the third serial measurement at ≥15 days.

### Clinical performance.

A receiver operating characteristic analysis was fitted to determine the best cut point supporting positive diagnoses from a group of 1,568 samples (188 positive samples) with an area under the curve (AUC) of 0.980 (95% CI, 0.960 to 0.990; *P* < 0.0001). The maximum Youden index occurred at a cut point of 9.4 (95% CI, >7.1 AU/ml to >12.1 AU/ml), with a sensitivity of 95% and a specificity of 97%.

**FIG 2:**
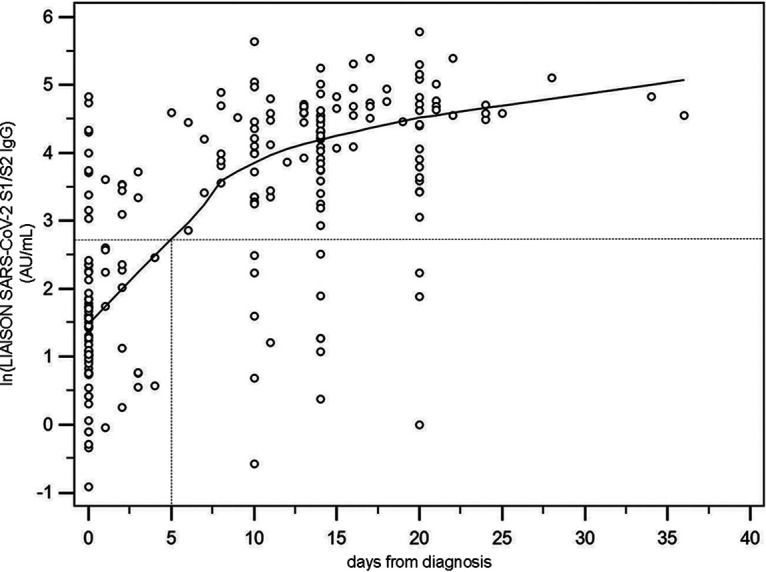
SARS-CoV-2 S1/S2 IgG measurements for 211 samples collected over the course of time from 84 patients at admission and variably thereafter up to 36 days. The upward trend was modeled using an exponential regression [ln(IgG) = *A* + *B* · exp(*C* · days)]. The parameter *A* (4.58) represents the upper limit to which the LIAISON SARS-CoV-2 S1/S2 IgG trends over time and corresponds to 98 AU/ml. *A* + *B* (1.63) is the value at time zero corresponding to 5.1 AU/ml on the original scale. The parameter *C* (−0.112) is the rate at which the curve moves up to the asymptote and corresponds to 1.1 AU/ml per day. The dotted horizontal line is 15 AU/ml, the positive cutoff the assay. The curve cuts this at 5 days, giving an estimate of the delay from diagnosis above which the patient is, on average, positive.

The clinical performance of the LIAISON SARS-CoV-2 S1/S2 IgG assay is shown in Table 3. The sensitivity was determined by investigating 211 samples collected longitudinally over the course of time from 84 patients at admission and thereafter collected variably up to 36 days. Infection with SARS-CoV-2 was confirmed by a positive RT-PCR test at the early phase of infection at the time of diagnosis. Logarithmic values of SARS-Cov-2 S1/S2 IgG are plotted over time with a fitted curve (Fig. 2), projecting estimations of 5 days for an average sample to reach 15 AU/ml, with 92.9% of the samples exceeding a threshold of 15 AU/ml by 5 or more days postdiagnosis. Table 3 compares the sensitivities and specificities consequent to the higher cutoff of 15 AU/ml currently suggested by the manufacturer’s instructions for use to a lower cutoff of 9 AU/ml. Diagnostic sensitivity with the lower cutoff is calculated at 33.3% for the early samples (≤5 days after diagnosis) and 91.3% for samples collected >5 days postdiagnosis. Diagnostic sensitivity with the higher cutoff drops to 22.6% for the early samples (≤5 days after diagnosis) and 88.2% for samples collected >5 days postdiagnosis. Conversely, specificity (from the testing of 1,140 stored residual laboratory samples routinely collected before the COVID-19 outbreak) increased slightly from 97.1% to 98.5% at the lower and higher cutoffs, respectively. Specificities evaluated using all negative samples were 97.0% and 98.1%.

**TABLE 3:** Clinical performance of the LIAISON SARS-CoV-2 S1/S2 IgG assay using 9 and 15 AU/ml as cutoffs based on RT-PCR diagnoses

Cutoff (AU/ml)	Sensitivity or specificity	Time period or test result	LIAISON SARS-CoV-2 S1/S2 IgG assay		
			% of positive samples	95% CI	No. of samples
9	Sensitivity	≤5 days	33.3	23.4–44.5	84
		>5 days	91.3	85.0–95.6	127
	Specificity	Pre-COVID-19	97.1	96.0–98.0	1,140
		All negative results	97.0	95.9–97.8	1,380
15	Sensitivity	≤5 days	22.6	14.2–33.0	84
		>5 days	88.2	81.3–93.2	127
	Specificity	Pre-COVID-19	98.5	97.6–99.1	1,140
		All negative results	98.1	97.2–98.8	1,380

### Comparison to samples with neutralizing titers.

**FIG 3:**
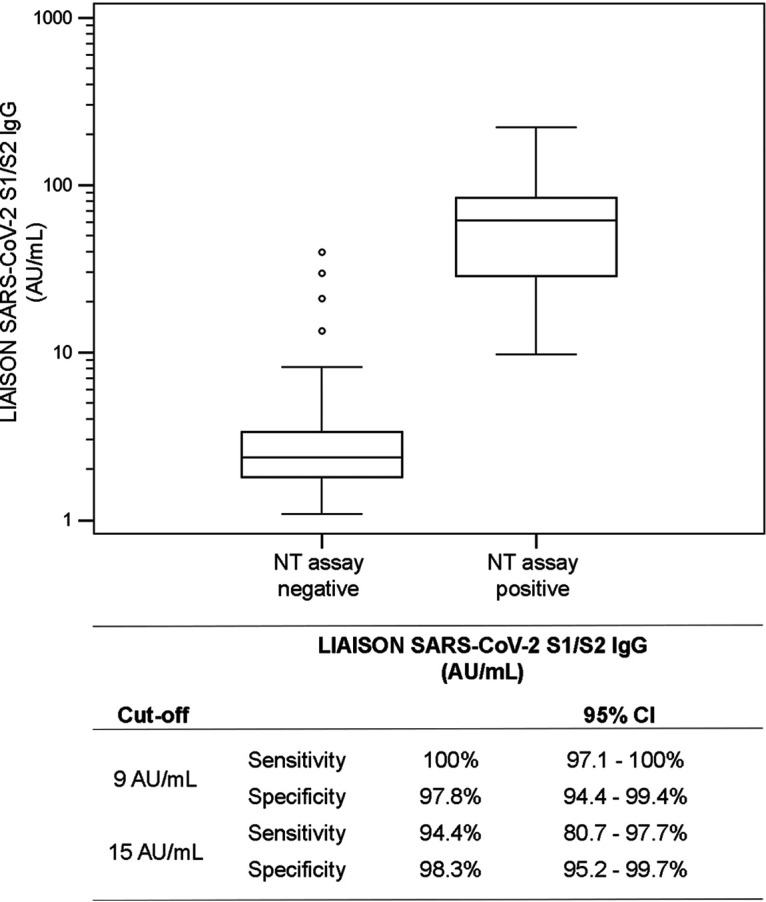
Distribution of the LIAISON SARS-CoV-2 S1/S2 IgG assay measurements compared to values for neutralization-positive (titers ≥ 1:40) and -negative (titers < 1:40) samples by the NT assay. Assay performance is presented specifically for neutralization using 9 and 15 AU/ml as cutoffs.

**FIG 4:**
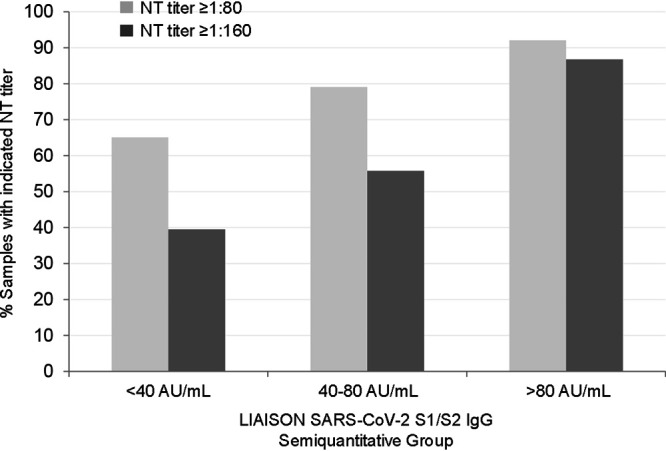
Relationship to and distribution of the LIAISON SARS-CoV-2 S1/S2 IgG assay levels versus NT dilutions. LIAISON SARS-CoV-2 S1/S2 IgG measurements were separated into 3 groups (<40 AU/ml, 40 to 80 AU/ml, and >80 AU/ml) and related to NT assay groups by titers: ≥1:160 (dark gray) or ≥1:80 (light gray). In the >80-AU/ml group, 33 of 38 samples have an NT assay titer of ≥1:160, while 35 of 38 samples have an NT assay titer of ≥1:80. Both titers are considered acceptable by FDA guidelines (12). The respective semiquantitative groups contain 43, 43, and 38 samples.

How the results of a neutralization (NT) assay compared to those of the LIAISON SARS-CoV-2 S1/S2 IgG assay was evaluated by testing 304 samples collected during the outbreak from subjects whose NT assay results were available; 180 were NT assay negative, and 124 were NT assay positive (titer > 1:40). Positive agreement was 94.4% (95% CI, 88.8% to 97.2%), and negative agreement was 97.8% (95% CI, 94.1% to 99.1%). The relationship between the LIAISON SARS-CoV-2 S1/S2 IgG assay and NT assay-negative or NT assay-positive samples portrays a nearly complete separation between the 2 groups, with medians of 2.4 AU/ml (95% CI, 2.2 to 2.6 AU/ml) and 61.8 AU/ml (95% CI, 50.3 to 70.7 AU/ml), respectively (Fig. 3). In Fig. 4, the LIAISON SARS-CoV-2 S1/S2 IgG assay’s measurements were separated into 3 semiquantitative groups (<40 AU/ml, 40 to 80 AU/ml, and >80 AU/ml) and related to NT assay titers of ≥1:160, which is the threshold recommended by the FDA guidelines for use in convalescent-phase blood transfusion. Thirty-nine percent (17/43), 56% (24/43), and 87% (33/38) of the samples, respectively, had NT assay titers of ≥1:160 (12). Furthermore, since the FDA guidelines also admit NT assay titers of ≥1:80 as acceptable, additional leeway is granted toward use of the LIAISON SARS-CoV-2 S1/S2 IgG assay to prescreen or assess blood donor samples for potential convalescent-phase plasma/serum therapy; 92% (35/38) and 79% (34/43) of the >80-AU/ml and 40- to 80-AU/ml groups, respectively, had NT assay titers of ≥1:80.

### Analytical performance.

The LIAISON SARS-CoV-2 S1/S2 assay was evaluated for intra-assay imprecision using 6 samples with moderate, low, or negative S1/S2 IgG levels over 5 days with multiple replicates and runs (*n* = 90). The average intra-assay imprecision was a 2.8% coefficient of variation (2.8%CV) (range, 2.0 to 3.4%CV), and total-assay imprecision averaged 3.2%CV (range, 2.7 to 3.9%CV).

Cross-reactivity with other coronaviruses was tested against 10 patient samples positive by their respective RT-PCR tests to other coronaviruses (HCoV-229E, HCoV-HKU1, HCoV-OC43, and HCoV-untyped) that maintained negative NT assay results for SARS-Cov-2. Their LIAISON values ranged from 1.81 to 7.09 AU/ml, with an average of 3.45 AU/ml, and fell far below both cut points of 9 and 15 AU/ml, indicating the absence of cross-reactivity with the other coronaviruses tested. Additionally, cross-reactivity was assessed in samples from patients with conditions caused by other viruses presenting with symptoms similar to those of SARS-CoV-2 infection, other infectious disease organisms, or atypical immune system activity (details can be found in Materials and Methods). Three out of 160 assessed specimens (1.9%) were positive with the LIAISON SARS-CoV-2 S1/S2 IgG assay (herpesvirus B, influenza A, and rheumatoid factor samples each had one positive call). Potentially interfering substances, such as triglycerides (3,000 mg/dl), cholesterol (400 mg/dl), hemoglobin (1,000 mg/dl), conjugated and unconjugated bilirubin (40 mg/dl), acetaminophen (500 mg/ml), and ibuprofen (500 mg/ml) showed no interference at the indicated concentrations. The LIAISON SARS-CoV-2 S1/S2 IgG assay demonstrated a negative bias of up to 16% in S1/S2 IgG-positive specimens with biotin concentrations above 3,500 ng/ml, a concentration 15-fold higher than that induced following ingestion of a 20-mg/day biotin supplement (13).

## DISCUSSION

SARS-Cov-2 is a single-strand, positive-sense RNA virus that is most closely related to SARS-CoV and other B lineage members of the β genogroup of coronaviruses (5, 14). Other readily recognized members of the coronavirus family include the extremely virulent Middle East respiratory syndrome (MERS) CoV and the less virulent OC43, HKU1, 229E, and NL63, HCoVs more commonly associated with the common cold in adults. Coronavirus RNA encodes four major categories of structural proteins, including membrane, envelope, nucleocapsid, and spike, which are referred to as M, E, N, and S, respectively. While N protein elicits cell-mediated immunity attributable to two predominant CD8 T cell epitopes (15), of the remaining three structural proteins, S protein is widely recognized as that most specific with regard to generating protective, neutralizing antibodies (16, 17).

The specificities reported from *in vitro* diagnostic immunoassays (18–20) are impacted greatly by the fidelity of preservation of both linear and conformational epitopes of the given analyte being measured for presentation to specific immunoglobulins within a patient’s serum sample (here, SARS-Cov-2 S1/S2). Specificity is frequently significantly compromised by the casual manner in which most ELISAs are fabricated. This is a consequence of the generally accepted means of passive adsorption of the target analyte protein to the plastic or nylon surfaces of microtiter plates. This procedure induces significant structural deformation and denaturation, with the consequent loss of native conformation, as well as occlusion of access to both conformational and linear epitopes beneath the protein stuck to the plastic titer plate’s surface. As explained in Materials and Methods, our system allows for optimal maintenance of spike protein conformation. Consequently, the LIAISON SARS-CoV-2 S1/S2 IgG assay rendered no false-positive results from NT assay-negative, RT-PCR-positive samples from related coronavirus members, and its performance is sensitive, specific, and precise as evaluated with >1,500 samples.

The use of convalescent-phase serum to treat subjects with acute SARS-Cov-2 infection has growing appeal for meeting the immediate challenges being imposed upon increasingly stressed health care systems, in light of the premature status of vaccines and the limited availability of effective antiviral therapeutic regimens (21–25). The LIAISON SARS-CoV-2 S1/S2 IgG assay was designed to detect IgG with neutralizing potential and is shown here to have very good sensitivity and specificity in identifying samples with positive neutralization titers. Furthermore, if used in a semiquantitative manner, higher LIAISON units are indicative of higher NT assay titers and provide a tool to prescreen large numbers of samples. While neutralization tests provide the recognized benchmark, they are not practical for large-scale implementation due to requirements for high biosecurity containment and the need for highly trained personnel to execute labor-intensive protocols. Here, independent from both of these constraints, clear separation of NT assay-negative samples from NT assay-positive samples was achieved. In fact, with 40- to 80-AU/ml levels measured by the LIAISON SARS-CoV-2 S1/S2 IgG assay, the probabilities of having neutralization titers of ≥1:80 and ≥1:160 were 79% and 56%, while with >80 AU/ml, the probability of having neutralization titers of ≥1:80 and ≥1:160 increased to 92% and 87%, respectively. This may be useful for the efficient screening of convalescent-phase plasma for safe therapeutic use.

The LIAISON SARS-CoV-2 S1/S2 IgG assay’s sensitivity increases significantly as the immune response matures, as one would expect for an IgG-based serology assay’s assessment of host responses to viral infection (Tables 2 and 3 and Fig. 2). Here, we report sensitivities of 33.3% at <5 days but of >91% at ≥5 days postadmission for samples from 104 Italian patients whose RT-PCR tests were positive at the time of diagnosis. The importance of choosing a cutoff that provides high sensitivity (9 AU/ml) versus one that provides low sensitivity but high specificity (15 AU/ml) is influenced by disease prevalence, reflected in positive and negative predictive values (PPV and NPV). When the intent is to use the assay for screening, a higher threshold may be desirable, whereas in high-prevalence environments, such as hospitals caring for high numbers of COVID-19 subjects, when the test is used to aid in diagnosis, the lower threshold of 9 AU/ml may be preferred. Serology tests are now being utilized to gain an initial assessment of infection prevalence, with reported numbers of ∼3% and ∼20% from California and New York, respectively (27). In California, a negative test with the LIAISON assay would have an accompanying NPV of >99.5%, and in New York City, it would have an NPV of >97.5%, regardless of the cutoff, indicating that staying at home and avoiding exposure is the best option. However, a positive test presents a quite different story; California’s PPV of 80%, derived from the higher cutoff despite the test being less sensitive overall, would provide a positive test result, affording more confidence in the subject’s true positivity, while the lower cutoff would present some ambiguity with regard to any subject’s real level of protection (PPV of 62%). In New York, however, regardless of the cutoff, the PPV of 89 to 95% affords a much greater degree of confidence that an individual has protective levels of antibody. When testing in a hospital setting, the lower cutoff may be preferable to ensure a higher NPV, even though the overall specificity may be decreased.

In conclusion, the automated LIAISON SARS-CoV-2 S1/S2 IgG assay brings efficient, sensitive, specific, and precise serological testing to the laboratory. Further, the assay is amenable for semiquantitative, efficient prescreening of samples for neutralizing antibody content for use in convalescent-phase plasma therapy.
